# Activity-dependent COX-2 proteolysis modulates aerobic respiration and proliferation in a prostaglandin-independent manner

**DOI:** 10.1016/j.isci.2024.111403

**Published:** 2024-11-17

**Authors:** Liat Hagit Hartal-Benishay, Sharon Tal, Amal Abd Elkader, Omar Ehsainieh, Ranin Srouji-Eid, Tali Lavy, Oded Kleifeld, Martin Mikl, Liza Barki-Harrington

**Affiliations:** 1 Department of Human Biology, Faculty of Natural Sciences, University of Haifa, Haifa 3103301 Israel; 2 Faculty of Biology, Technion - Israel Institute of Technology, Haifa 3200003, Israel

**Keywords:** Biomolecules, Molecular biology, Proteomics

## Abstract

Cyclooxygenase-2 (COX-2) catalyzes the oxidation of arachidonic acid (AA) into a single product that is the source of all prostaglandins (PGs), ligands of multiple pro-inflammatory pathways. AA catalysis results in suicide inactivation, rendering the enzyme catalytically inactive. Here, we report that catalytic activity also leads to controlled cleavage of COX-2, an event that is differentially regulated by fatty acids, and blocked by COX inhibitors. We also find COX-2 fragments in human colon tumors. Using mass spectrometry, we identified two adjacent cleavage points within the catalytic domain, which give rise to COX-2 fragments that are catalytically inactive and localize to different cellular compartments. Expression of one of these fragments in cells significantly reduced mitochondrial function, increased lactate production, and enhanced proliferation. We propose that in addition to its role in generating PGs, COX-2 has PG-independent cellular functions that may account for its complex role in proliferative diseases and chronic inflammation.

## Highlights

• Catalytic activity of COX-2 leads to its controlled proteolysis
• Catalytically inactive COX-2 fragments migrate to nuclear compartments
• A COX-2 cleavage product reduces aerobic respiration and increases proliferation
• COX-2 plays dual, catalytic and non-catalytic roles in cell function

## Graphical abstract

**Figure:**
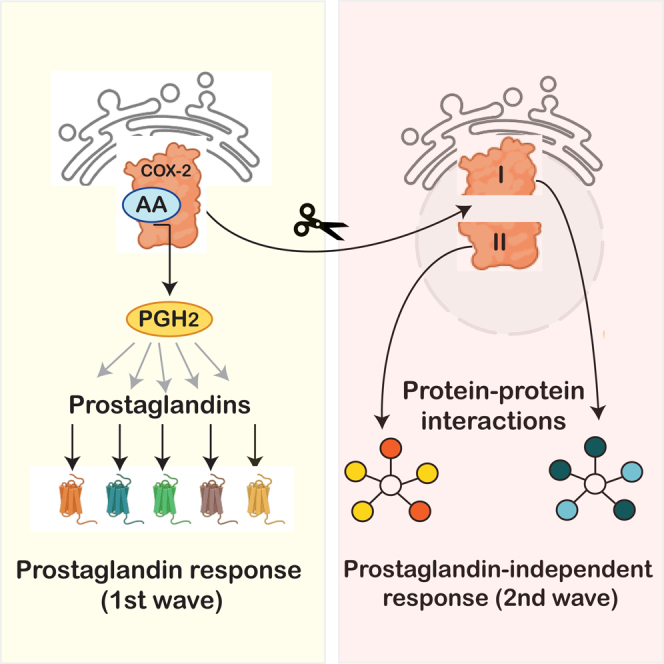

## Introduction

Cyclooxygenase-2 (COX-2) is an enzyme that catalyzes the rate-limiting step in the conversion of arachidonic acid (AA) to prostaglandins (PGs)—bioactive lipids that play central roles in inflammation. Except for several tissues where it is constitutively expressed (e.g., kidney, brain, endothelial cells), COX-2 expression is usually low, and its levels are rapidly upregulated in response to a wide range of inflammatory and pathological signals, thus producing the PGs that mediate a major part of inflammation.^1^

COX-2 catalyzes the oxidation of AA into prostaglandin H_2_ (PGH_2_), the source of all subsequent PGs, by a two-step redox reaction, which occurs in two structurally and functionally interconnected sites of the catalytic domain of the protein.^2^^,^^3^ The first is a cyclooxygenase (COX) reaction, which occurs in a hydrophobic channel in the core of the enzyme where PG endoperoxide G_2_ (PGG_2_) is generated. The subsequent peroxidase (POX) reaction occurs at a heme-containing active site located near the protein surface where PGG_2_ is reduced to PGH_2_. Specific PG synthases then catalyze PGH_2_ into PGs (PGE_2_, PGD_2_, PGI_2_, and PGF_1α_) and thromboxane (TXA_2_), all of which activate multiple signaling pathways by binding to G protein-coupled receptors.^2^^,^^4^ The COX-2 enzyme is an obligatory dimer, consisting of two identical monomers, each containing the same active site for AA catalysis. However, only one of the two active sites catalyzes AA,^5^^,^^6^ while the other serves as an allosteric modulator of the active site. The rate of catalysis is affected by fatty acids (FAs), non-steroidal anti-inflammatory drugs (NSAIDs), and selective COX-2 inhibitors that either compete with AA in the catalytic site or modulate its activity by binding to the allosteric site.^7^^,^^8^^,^^9^^,^^10^

Free radicals formed during catalysis cause COX-2 to quickly become catalytically inactive, by a mechanism of substrate-dependent suicide inactivation.^2^^,^^3^^,^^11^ Interestingly, whereas the cellular proteasome regularly degrades the quiescent COX-2 that has not been exposed to AA, the fate of the catalytically inactive protein is largely unknown.^12^^,^^13^ We have previously shown that some of the substrate-inactivated COX-2 is discarded from the cells by exosomes.^14^ Now we show that AA-stimulation also causes the COX-2 protein to undergo limited proteolysis and that one of the resulting fragments inhibits aerobic respiration and increases proliferation in a PG-independent manner.

## Results

### A fragment of COX-2 appears following its stimulation with AA

**Figure 1:**
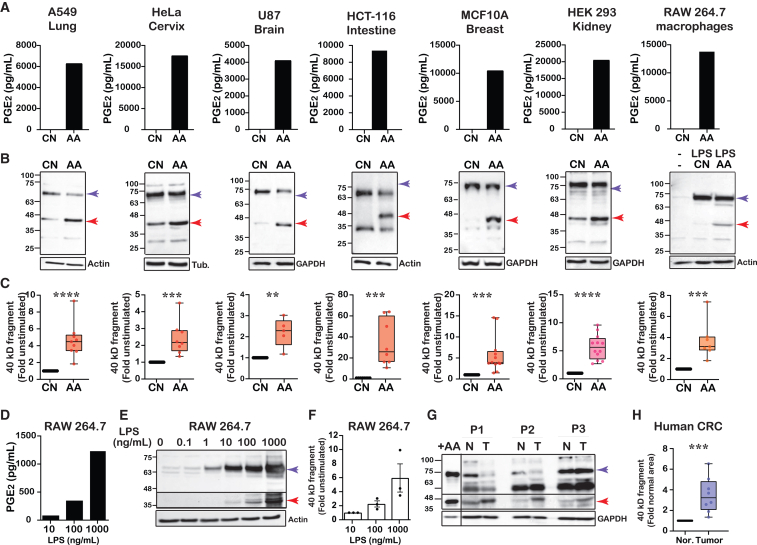
A fragment of COX-2 appears following its stimulation with AA (A) Quantification of PGE_2_ levels produced by stimulation of different cell lines, before and after stimulation with 50 μM arachidonic acid (AA) for 30 min: A549 (human lung adenocarcinoma, endogenous expression), HeLa (human cervical adenocarcinoma, endogenous expression), U87 (human brain glioma, endogenous expression), HCT-116 (human colorectal adenocarcinoma, endogenous expression), MCF10A (human breast, fibrocystic disease, endogenous expression), HEK 293 (human embryonic kidney, overexpressed by transfection), RAW 264.7 (mouse macrophages, LPS-induced). (B) Representative western blots of full-length (F.L.) COX-2 (purple arrow) and the 40 kD fragment (red arrow) from the cell lines in (A), with and without stimulation with 50 μM AA for 30 min. (C) Quantification of the change in the levels of the 40 kD immunoreactive band following AA stimulation: A549 (*n* = 9), HeLa (*n* = 8), U87 (*n* = 5), HCT-116 (*n* = 8), and MCF10A (*n* = 11), HEK 293 (*n* = 12). RAW 264.7 cells were treated overnight with 10 ng/mL before stimulation with AA (*n* = 6). (D) Quantification of PGE_2_ levels produced by stimulation of RAW 264.7 cells for 24 h with different concentrations of LPS. (E) Representative western blot of RAW 264.7 cells treated for 24 h with 0.1–1000 ng/mL LPS. F.L. COX-2 (purple arrow) and the 40 kD fragment (red arrow) are presented separately for better detection purposes. (F) Quantification of the LPS dose response of RAW 264.7 in (E) (*n* = 3). (G) Representative samples from a tumor (T) and surrounding normal tissues (N) obtained from three colorectal cancer (CRC) patients (P1-3), ran alongside a sample of COX-2 expressing HEK 293 cells stimulated with AA. The line marks a separate exposure of the control from the CRC samples. The lower 40 kD band was separately exposed for better detection (See “Study limitations). (H) Quantification of *n* = 8 CRC samples. Statistical analysis was performed using the Wilcoxon test. Quantification of the protein levels of the COX-2 fragment was done after normalization by GAPDH or actin. Data are represented as mean ± SD. Statistical significance was assessed by Student’s t test. ^∗^*p* < 0.05, ^∗∗^*p* < 0.01, ^∗∗∗^*p* < 0.001, and ^∗∗∗∗^*p* < 0.0001.

The full-length (F.L.) COX-2 is a protein of ∼72 kD. We have previously shown that a brief stimulation of HEK 293 cells overexpressing COX-2 with AA, causes the appearance of a smaller COX-2 immunoreactive band of ∼40 kD.^14^ To determine whether this is a general phenotype of COX-2, we stimulated multiple human cell lines with endogenous COX-2 expression, with AA, and probed the samples with an anti-COX-2 antibody directed against the N-terminal of the protein. As depicted in Figure 1A, stimulation of all cell lines with AA resulted in the generation of PGE_2_, one of the PG products of COX-2 activity, confirming that COX-2 in these cells is catalytically active. In parallel, AA stimulation also caused the appearance of a lower ∼40 KD COX-2 immunoreactive band in all cell lines examined (Figures 1B and 1C). Some cells (e.g., HeLa, HCT-116, MCF10A) also expressed additional COX-2 immunoreactive bands that were not affected by the presence of AA. An immunoreactive band of the same size was observed after AA stimulation in the murine-derived RAW 264.7 macrophages following induction of COX-2 by lipopolysaccharide (LPS), and in HEK 293 cells with exogenous expression of COX-2, suggesting that this is a general phenomenon that is not restricted to a certain cell line or species or to whether COX-2 is endogenously or ectopically expressed.

The effect of AA stimulation on the appearance of the lower COX-2 band was dose-dependent and appeared with concentrations as low as 0.5–5 μM AA (Figure S1). However, we sought to determine whether induction of AA release by pro-inflammatory agents as occurs physiologically, also results in the same effect. As depicted in Figure 1D, exposure of RAW 264.7 cells to increasing concentrations of LPS resulted in a dose-dependent elevation in PGE_2_ levels. Although LPS exposure yielded significantly lower PGE_2_ levels compared to direct stimulation with high levels of AA (Figure 1A), it caused the appearance of the lower COX-2 immunoreactive band in a dose-dependent manner (Figures 1E and 1F).

Finally, we searched for the presence of lower COX-2 immunoreactive bands *in vivo*. For this, we examined its expression in a small cohort of eight samples of colorectal tumors of patients diagnosed with low or moderate colorectal adenocarcinoma.^15^ As depicted in Figure 1G, cancerous samples also showed several COX-2 immunoreactive bands that differed between the tumor and the surrounding clean margins. One of the bands that were elevated in the tumor samples was of ∼40 kD and migrated to the same distance as the control sample of COX-2-expressing cells stimulated with AA (Figure 1G, lane 1). The sample size of the patient cohort is too small to make any meaningful correlations (e.g., sex, pathology, severity, prognosis, etc.), and the exact identity of all of the smaller COX-2 immunoreactive bands in each sample is currently unknown. However, their existence *in vivo* supported the rationale to study the possible unknown functions of COX-2 fragments in the cell.

### The appearance of the COX-2 fragment requires enzymatic activity

**Figure 2:**
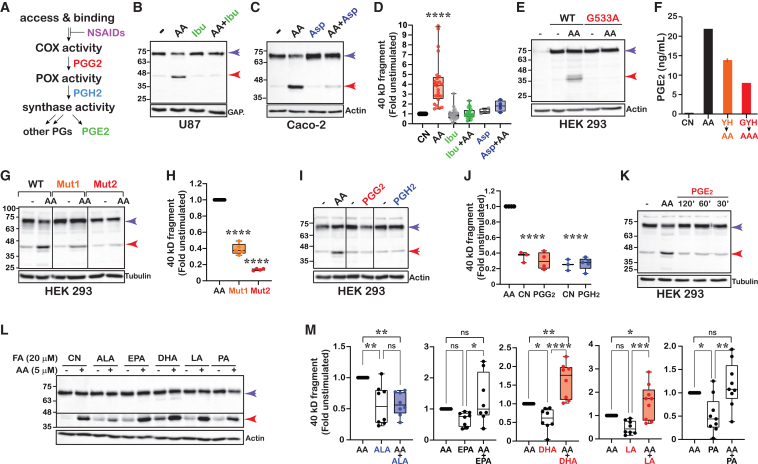
The appearance of the COX-2 fragment requires enzymatic activity (A) Scheme of the different stages of the prostaglandin biosynthesis pathway (B) Western blot of U87 cells stimulated with AA in the absence or presence of ibuprofen (Ibu). (C) Western blot of Caco-2 cells stimulated with AA in the absence or presence of aspirin (Asp). (D) Box and whisker plot illustrating the quantification of average changes in the levels of the COX-2 fragment in response to COX inhibitors. A total of *n* = 24 consisting of data obtained from U87 (*n* = 4), A549 (*n* = 6), HeLa (*n* = 2), RAW 264.7 (*n* = 3), Caco-2 (*n* = 5), HCT-116 (*n* = 4) for ibuprofen, or aspirin (Caco-2, *n* = 4). (E) Western blot depicting the response of HEK 293 cells expressing WT or G533A COX-2 to AA stimulation. (F) Quantification of PGE_2_ levels produced by stimulation of HEK 293 cells expressing WT and COX-2 mutants: Mut 1 (L352A, S353A, Y355A, H356A, orange), or Mut 2 (L352A, S353A, G354A, Y355A, H356A) (*n* = 2). (G) Western blot depicting the response of HEK 293 cells expressing either WT COX-2 or the mutants in (F), to AA stimulation. (H) Quantification of the COX-2 fragment levels in (G) after normalization by GAPDH (*n* = 4). (I) Western blot depicting the response of COX-2-expressing HEK 293 cells to stimulation with 5 μM AA, PGG_2_, or PGH_2_. (J) Quantification of the COX-2 fragment levels in (I) after normalization by actin (*n* = 5). (K) Western blot of COX-2 expressing HEK 293 cells, treated with either AA or PGE_2_ at different time points. (L) Representative western blot of RAW 264.7 cells stimulated overnight with 10 ng/mL LPS followed by treatment with 20 μM of different fatty acids for 30 min before stimulation with 5 μM AA. (M) Quantification of the COX-2 fragment levels in cell lines treated as in (L): HEK 293 (*n* = 1), Caco-2 (*n* = 2), A549 (*n* = 2), RAW 264.7 (*n* = 4). Data are represented as mean ± SD. Statistical significance was assessed by Student’s t test or one-way ANOVA. ^∗^*p* < 0.05, ^∗∗^*p* < 0.01, ^∗∗∗^*p* < 0.001, and ^∗∗∗∗^*p* < 0.0001.

Catalysis of AA by COX-2 involves binding of AA to the catalytic domain and a two-step catalytic reaction (Figure 2A).^16^ To test whether AA binding to the protein is necessary to induce the formation of the COX-2 fragment, we pretreated the cells with reversible or irreversible inhibitors of COX-2 (ibuprofen and aspirin, respectively), both of which inhibit catalytic activity by preventing AA access to the catalytic sites.^2^ As depicted in Figures 2B–2D, both compounds prevented the AA-mediated appearance of the lower COX-2 immunoreactive band, as did a COX-2 mutant that binds AA but shows minimal enzymatic activity compared to the native enzyme (G533A COX-2)^4^ (Figure 2E). A productive binding mode of AA to the enzyme is obtained when it is positioned such that the carboxylate lies near amino acids R120 and Y355, at the opening of the cyclooxygenase channel.^17^ We next introduced point mutations to Y355 and its vicinity and explored the effect on the formation of the COX-2 fragment. These mutations caused a severe reduction in the catalytic activity of COX-2 as measured by its ability to generate PGE_2_ (Figure 2F), which was accompanied by a significant decrease in the generation of the COX-2 fragment (Figures 2G and 2H).

Amino acid Y355 was later identified as one of the cleavage points of COX-2, therefore mutating this site does not indicate whether the effect of AA is lost due to lack of proper binding, or elimination of a cleavage site. To further localize the AA effect on fragment formation, we compared its effect to that of the two products of catalysis, namely, PGG_2_ (step 1) and PGH_2_ (step 2) (Figure 2A). Remarkably, only AA caused the appearance of the COX-2 fragment, while PGG_2_ and PGH_2_ failed to do so (Figures 2I and 2J). Finally, treatment of COX-2-expressing cells with PGE_2_, one of the most abundant products of AA catalysis did not cause the appearance of the COX-2 fragment (Figure 2K), suggesting that cleavage is probably not mediated by immediate downstream PG signaling. Together, these data indicate that formation of the COX-2 fragment requires productive AA binding and perhaps COX activity, but is not dependent upon the later stages of catalysis (Figure 2A).

The rate of AA catalysis by COX-2 was previously shown to be differentially modulated by the binding of substrate- and non-substrate FAs.^5^^,^^7^^,^^10^ To determine if FAs also affect the appearance of the COX-2 fragment, we treated COX-2-expressing cells with 20 μM saturated and unsaturated (ω3 and ω6) FAs before stimulation with 5 μM AA (4:1). As depicted in Figures 2L and 2M, eicosapentaenoic acid (EPA, 20:5ω3) and palmitic acid (PA, 16:0) acid had no significant effect on the appearance of the COX-2 fragment, α linolenic acid (ALA, 18:3ω3) reduced the AA effect and linoleic acid (LA, 18:2ω6) and docosahexaenoic acid (DHA, 22:6ω3) increased it. These findings suggest that the formation of the COX-2 fragment following enzymatic activity is a physiologically relevant event, which is differentially affected by the content of FAs present and their ratio to AA.

**Figure 3:**
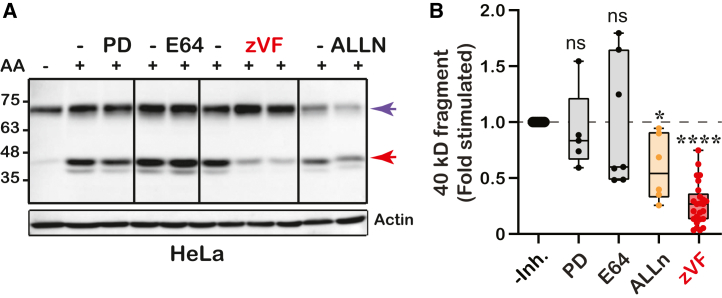
The formation of the COX-2 fragment is dependent on proteolysis (A) A representative blot of HeLa cells treated with the protease inhibitors PD150606 (PD, 50 μM), E64D (10 μM), zVF (50 and 100 μM), and ALLN (10 μM) overnight, before AA stimulation. (B) Quantification of COX-2 fragment levels in (A) after normalization by GAPDH or actin in response following treatment with protease inhibitors: PD150606 (*n* = 5), E64 (*n* = 7), ALLN (*n* = 6), zVF (*n* = 25). Data are represented as mean ± SD. Statistical analysis was assessed by One-way ANOVA ^∗∗∗∗^*p* < 0.0001, with Dunnett’s multiple comparisons post-test.

Our data indicates that the appearance of the COX-2 fragment is a post-translational event that may be carried out by proteolysis. Previous studies had indicated that cleavage of the F.L. COX-2 is reduced by different cysteine protease inhibitors, among which calpain inhibitors had the strongest effect.^18^^,^^19^ To test whether calpain, a calcium-dependent cysteine protease is also involved in AA-mediated generation of the COX-2 fragment, we tested its formation in the presence of an array of general cysteine protease inhibitors and selective calpain inhibitors. As shown in Figure 3A, E64 (a broad-spectrum cysteine protease inhibitor)^19^ and PD150606 (a selective calpain inhibitor)^20^ did not affect the AA-mediated formation of the COX-2 fragment. Application of ALLN (calpain inhibitor I) reduced the appearance of the fragment by approximately 60%, and Z-Val Phe-OH (zVF, Calpain III), a potent cell-permeable calpain I and II inhibitor, had the most profound effect, inhibiting approximately 75% of cleavage (Figure 3B).

### Identification and ectopic expression of COX-2 fragments

**Figure 4:**
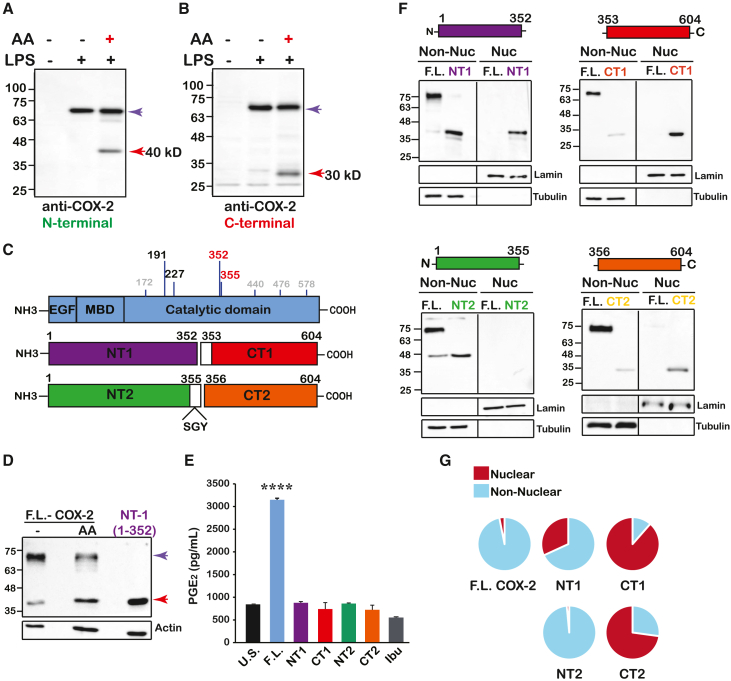
Identification and ectopic expression of COX-2 fragments (A and B) Western blots of RAW 264.7 cells were stimulated overnight with LPS before exposure to AA using antibodies against the amino- (A) and carboxy- (B) termini of COX-2. (C) Schematic depiction of the cleavage sites identified by LC-MS/MS in RAW 264.7 cells and the overlapping amino acids in homo sapiens (Top drawing). Schematic representations of the putative cleavage products that were introduced into cells. NT1 = 1–352, CT1 = 353–604 (middle drawing), and NT2 = 1–355 and CT2 = 356–604 (bottom drawing). (D) Western blot depicting NT1 relative to AA-stimulated COX-2 in HEK 293 cells. (E) Quantification of PGE_2_ levels produced by HEK 293 cells expressing WT COX-2 or the different fragments. U.S. represents cells expressing WT COX-2 without stimulation, and Ibu represents cells treated with ibuprofen for 30 min before AA stimulation, (*n* = 2). Data are presented as mean + SD. Statistical significance was assessed by One-way-Anova. ∗∗∗∗*p* < 0.0001. (F) Representative immunoblots of nuclear (Nuc) and non-nuclear (Non-Nuc) fractions of HEK293 cells expressing F.L. COX-2 or each of the four fragments. (G) Pie charts quantifications of COX-2 or fragments localization after normalization of nuclear fractions to lamin B and non-nuclear fractions to tubulin. NT1 (*n* = 7), CT1 (*n* = 5), NT2 (*n* = 4), CT2 (*n* = 7).

Given the previously findings, we next sought to identify AA-mediated cleavage points in COX-2. For this purpose, we stimulated RAW 264.7 cells with LPS to induce COX-2 expression, followed by exposure to AA to induce cleavage. Samples were then separated by SDS-PAGE, and proteins were extracted and subjected to liquid chromatography with tandem mass spectrometry (LC-MS/MS) analysis. Total lysates from the same experiments were analyzed by western blot in parallel, to confirm the presence of the fragment. Probing the membranes with anti-COX-2 against the N-terminal of COX-2 identified the F.L. COX-2 as well as the additional ∼40 kD band that was detected in the other cell lines (Figures 4A and 41). The use of an additional antibody directed against the C-terminal of COX-2 detected the F.L. form and a prominent band of ∼30 kD (Figure 4B). Given that the size of F.L. COX-2 is ∼72 kD, these results suggest that at least one cleavage point is present roughly in the middle of the protein, one which gives rise to two complementary fragments of ∼40 and ∼30 kD.

Analysis of three independent LC-MS/MS experiments yielded eight putative cleavage points, four of which were positioned at amino acids that are identical to those of human COX-2 (191, 227, 352, and 355, Figure 4C). Interestingly, the estimated mass of two cleavage products that were very close to one another (positions 352–353 and 355–356, marked in red) was ∼40 kD (14–352/14-355) and 30 kD (353–604 and 355–604) respectively, which is similar to the estimated mass of the fragments obtained by western blot (Figures 4A and 4B). To confirm this, we ectopically expressed one of the putative fragments (14–352, NT1 herein) in HEK 293 cells, and ran the sample alongside a sample of AA-stimulated COX-2. As shown in Figure 4D, NT1 migrated to the same distance as the 40 kD COX-2 immunoreactive fragment that appeared following AA stimulation.

Our data show that cleavage of COX-2 is a post-translational event that must be preceded by enzymatic activity (Figure 2). Therefore, studying the possible effects of the AA-dependent fragments cannot be done simply by activation of COX-2 with AA, since this will yield a mixed response of PG-dependent and independent effects. To separate between the two, we cloned the sequences of two pairs of putative COX-2 fragments that complement each other to the size of F.L. COX-2: NT1 (amino acids, aa 14–352, Figure 4D) and its complementary CT1 (aa 353–604) and NT2 (aa 14–355) and its complementary CT2 (aa 356–604), and expressed each one ectopically. We then confirmed that in contrast to F.L. COX-2, none of these fragments can generate PGE_2_, in response to AA stimulation, indicating that they lack enzymatic activity (Figure 4E).

The COX-2 homodimer is associated with the ER and nuclear membranes by four amphipathic helices of its membrane binding domain (MBD). These chains surround the opening of the cyclooxygenase channel through which AA enters the COX site.^21^ Since amino acids 352–353 are located near the opening of the cyclooxygenase channel ^17^(Figure S2), we hypothesized that loss of the MBD motif due to cleavage may result in changes in the cellular localization of some of the fragments, particularly CT1 and CT2, compared to that of the F.L. protein. To test this, we examined the cellular localization of the F.L. COX-2 and each of the four fragments. As depicted in Figures 4F and 4G, F.L. COX-2 appears almost entirely in the non-nuclear fractions (97%) as indicated by staining with antibodies against both the N- and C-termini (left lane of all images in Figure 4F). The same is true for fragment NT2 (99%) but not for NT1, 32% of which is found in nuclear fractions (Figure 4F top left panel; Figure 4G). In marked contrast to F.L. and NT fragments, both CT1 and CT2 localize primarily to the nuclear fractions, with CT1 localizing almost entirely to that fraction (90% and 73%, respectively, Figures 4F and 4G).

### CT1-COX-2 reduces mitochondrial function and increases lactate production and proliferation

**Figure 5:**
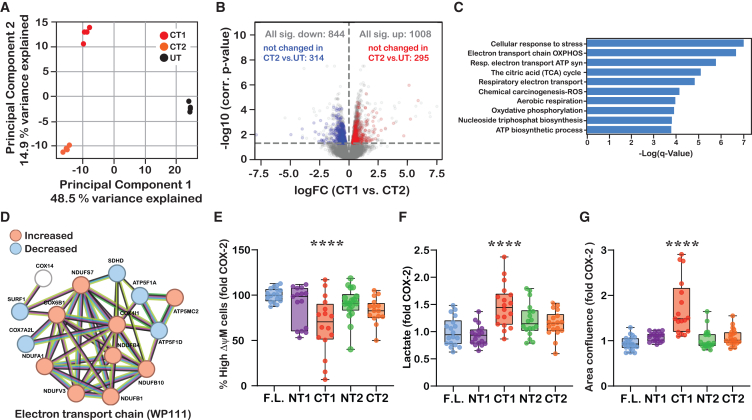
CT1-COX-2 reduces mitochondrial function and increases lactate production (A) Scatterplot showing the first two principal components from a PCA based on normalized and standardized expression values of all genes covered by at least 10 reads in all samples. (B) Volcano plot depicting the differential expression of genes between CT1-and CT2-expressing cells. The horizontal dashed line indicates the significance cutoff (adjusted *p*-value<0.05). Red and blue data points indicate genes that change between CT1-and CT2-expressing cells but do not exhibit a significant increase or decrease in CT2-expressing compared to control cells. (C) Top 10 most significantly enriched functional groups (as determined using Metascape) among the upregulated genes in CT1-compared to CT2-expressing cells, which showed no significant change between CT2-expressing and control cells. (D) STRING analysis of a DE gene set showing a minimum interaction score of highest confidence (0.9). (E) Box and whiskers plot describing the effect of COX-2 fragments on mitochondrial function, normalized to WT COX-2: F.L. COX-2 (*n* = 19), NT1 (*n* = 15), CT1 (*n* = 16), NT2 (*n* = 16), CT2 (*n* = 15). One-Way ANOVA, ∗∗∗*p* < 0.001 vs. COX-2 (Dunnett’s multiple comparisons post-test). (F) Box and whiskers plot describing the effect of COX-2 fragments on lactate production, normalized to WT COX-2: F.L. COX-2 (*n* = 18), NT1 (*n* = 17), CT1 (*n* = 18), NT2 (*n* = 18) CT2 (*n* = 18). One-Way ANOVA, ∗∗∗*p* < 0.001, 001 vs. COX-2 (Dunnett’s multiple comparisons post-test). (G) Box and whiskers plot describing the effect of COX-2 fragments on proliferation, normalized to WT COX-2: F.L. COX-2 (*n* = 20), NT1 (*n* = 20), CT1 (*n* = 17), NT2 (*n* = 21) CT2 (*n* = 21).

The marked differences in localization of CT1 and CT2 compared to F.L. COX-2 led us to postulate that they may interact with proteins in different cellular pathways and modulate their function by altering gene expression. To test this hypothesis, we sequenced the transcriptomes of control (untransfected, UT) and cells with stable expression of CT1 or CT2. A principal component analysis (PCA) of the resulting gene-level expression measurements revealed that both CT1 and CT2 exhibit different transcriptomic profiles compared to the control cells, as well as to each other (Figure 5A). Analysis of the RNA-seq data identified a total of 1852 genes that were differentially expressed (DE) between CT1 and CT2, (1008 genes, 54% upregulated (Table S1) and 844, 46% downregulated (Table S2); Benjamini-Hochberg adjusted *p* < 0.05) (Figure 5B), even though the two CT fragments differ only in the additional three N-terminal amino acids of CT1. To select for effects that were specific to the longer CT1 fragment that was almost exclusively found in nuclear fractions, we performed an additional filtering step for genes that also changed between CT2 and UT cells, which resulted in a list of 608 genes that were uniquely affected by the expression of CT1, 295 (49%) of which were upregulated (Table S3) and 313 (51%) were downregulated (Benjamini-Hochberg adjusted *p* < 0.05) (Table S4).

We next used Metascape^22^ to identify the pathways that are enriched among the DE genes (Tables S3 and S4). This analysis revealed a significant enrichment of genes belonging to cellular aerobic respiration among the genes upregulated in CT1-expressing cells (e.g., electron transport chain OXPHOS system in mitochondria, WP111, q = 2.0∗10^−7^; respiratory electron transport, R-HAS-163200, q = 1.5∗10^−6^; aerobic respiration, GO: 0009060, q = 1.1∗10^−4^) (Figures 5C and Table S5). The genes downregulated in CT1-expressing cells showed less significant enrichment for functional groups (Figure S3 and Table S6). Application of the STRING database^23^ on all DE genes revealed a network of 17 genes belonging to the electron transport chain (WP111 electron transport chain OXPHOS system in mitochondria, Figure 5D). Interestingly, the effect on the electron transport chain was mixed: genes belonging to complex I (NADH-Ubiquinone oxidoreductase) were all elevated, whereas those belonging to complex II and V (succinate-ubiquinone oxidoreductase, and ATP synthase, respectively) were downregulated. Genes belonging to complex IV showed a mixed effect where some genes were upregulated while others were downregulated (Figure 5D).

Given the implications for an effect on aerobic respiration, we next measured the effect of all COX-2 fragments on mitochondrial membrane potential using tetramethylrhodamine ethyl ester (TMRE).^24^ Remarkably, only cells that expressed CT1 presented a significant reduction in the levels of high-membrane potential mitochondria (Figure 5E). Reduced aerobic respiration can potentially lead to the generation of ATP by the anaerobic glycolysis pathway, which leads to an increase in lactate production. We therefore measured lactate levels in the supernatant of cells expressing the four fragments. The data depicted in Figure 5F confirmed that compared to cells expressing F.L. COX-2 only cells that expressed CT1 exhibited elevated lactate levels.

A reduction in the activity of oxidative phosphorylation despite the presence of oxygen is one of the hallmarks of rewiring of cancer cell metabolism. This phenomenon termed the Warburg effect, is characterized by a significant increase in lactate production due to the conversion of pyruvate to lactate instead of its metabolism by the mitochondria.^25^ Given the effect of CT1 on mitochondrial function and lactate production, we next measured the effect of COX-2 and its fragments on the rate of cell proliferation. As depicted in Figure 5G, the only COX-2 fragment that caused a consistent and significant increase in proliferation rate was CT1.

## Discussion

The main finding of the present study is that following AA-induced catalytic activity, COX-2 undergoes limited proteolysis, and at least one of the resulting fragments affects aerobic respiration and rate of proliferation. Importantly, the study shows that COX-2 cleavage occurs by direct stimulation of COX-2 with AA, as well as by indirect release of AA by LPS (Figure 1). Together with the evidence for increased COX-2 fragments in human tumors, and regulation of cleavage by FAs, these results point to a physiological role of COX-2 cleavage products. Hence, we propose that COX-2 fulfills two different roles in the cell. The first is its undisputed role as a central enzyme that generates soluble ligands of inflammation, and the second is a PG-independent role that is mediated through interactions of COX-2 fragments with different cellular pathways.

The structure and function of the COX-2 enzyme have been studied in-depth, providing detailed information regarding the kinetics of AA catalysis, and interactions of FAs and COX inhibitors with its catalytic and allosteric sites.^4^^,^^7^^,^^8^^,^^10^ In terms of its expression, COX-2 is an immediate-early gene that is rapidly and massively upregulated to meet the high demand for PGs during inflammation, and elevated COX-2 levels are hallmarks of chronic inflammation and many types of malignancies.^26^ Therefore, the majority of the high-volume literature regarding COX-2 focuses on its transcriptional regulation^1^ and on pharmacological means to inhibit its enzymatic activity.^27^ In contrast, much less is known about pathways that clear COX-2 from the cell. Previous studies have indicated that while in the absence of AA, COX-2 is degraded in the cellular proteasome, COX-2 which was catalytically active is no longer degraded there.^12^^,^^13^ This is a very important matter that is largely overlooked, especially since one of the most important properties of COX-2 is that its ability to generate PGs is limited by self-induced suicide inactivation occurring within seconds of activation.^28^ Previously, we demonstrated that part of the substrate-inactivated protein is discarded from the cells by exosomes.^14^ We further bridge the gap by showing that COX-2 is also subjected to controlled cleavage, which generates fragments that have secondary, non-catalytic effects.

COX-2 was previously shown to undergo proteolysis into discrete fragments following exposure of human synovial fibroblasts to pro-inflammatory cytokines.^19^ Using an anti-pan-human COX-2 with an undisclosed epitope, the researchers identified four COX-2 fragments, two of which are similar to those identified by us (44–42 and 34–36 kD). Using the same antibody against the C-terminus of COX-2 as we have (Figure 4B), they also identified a fragment of 30 kD, which like CT1 and CT2 (Figures 4F and 4G), localizes to nuclear and mitochondrial-lysosomal fractions. The similarity between the sizes of fragments in both studies is probably because the cytokine IL-1β causes AA release,^29^ much like we observed with LPS or direct AA stimulation (Figure 1). However, while Mancini et al. ascribe a role for proteolysis in protein maturation, we show that the putative fragments completely lack enzymatic activity, thus suggesting that they affect the cell in a PG-independent manner.^19^ These results are supported by our previously published finding that COX-2, which lacks the epidermal growth factor (EGF) and MBD domains (ΔCOX-2) is catalytically inactive.^30^

Given the presence of the same COX-2 fragments in several independent MS experiments, the lack of reports on protein breakage by tyrosyl radicals, and the sensitivity to different chemical protease inhibitors, it is most likely that COX-2 is cleaved by proteolysis. The search for protease/s that cleaves COX-2 is challenging. Whereas the F.L. unstimulated COX-2 is degraded in the proteasome, it no longer does so after catalytic activity.^13^ Since the putative cleavage site in our study lies at the surface of the protein,^17^ and given earlier indications,^18^^,^^19^ we examined calpain as a possible candidate for COX-2 proteolysis. Calpain was shown to degrade COX-2 *in vitro*,^18^ and indeed calpain inhibitors such as the calpain III inhibitor zVF significantly decreased the appearance of the 40 kD fragment (Figures 3A and 3B). However, zVF also targets γ-secretase,^31^ and even though it inhibited the majority of cleavage, it did not obliterate it, even in the presence of a cycloheximide that inhibits *de novo* synthesis (not shown). Therefore, the identity of the protease/s that cleaves COX-2 remains to be determined.

Multiple lines of evidence, both in cell lines and *in vivo*, indicate that COX-2 is involved in the initiation, promotion, and progression of cancer,^32^^,^^33^ and many studies show that elevated expression of COX-2 is strongly associated with a poor prognostic outcome.^34^^,^^35^^,^^36^ The role of COX-2 in tumorigenesis is attributed to the production of PGs that enhance proliferation by stimulation of their respective receptors^37^^,^^38^ thereby activating downstream signaling pathways that are associated with regulation of the cell cycle and proliferation.^39^^,^^40^ While there is a general agreement that inhibition of COX-2 is advantageous in cancer prevention,^41^^,^^42^^,^^43^^,^^44^^,^^45^^,^^46^^,^^47^ the efficiency of these drugs after disease onset is less straightforward. Several comprehensive studies showed that combining chemotherapy with COX-2 inhibitors in treating human cancers, is significantly beneficial in reducing tumor burden,^32^^,^^48^ while other large-scale ones found that celecoxib-combined therapy failed to improve disease-free survival or progression-free survival, and had no effect on pathological complete response.^49^ Clinical trials in malignancies where COX-2 expression correlates with poor prognosis (e.g., lung, breast, glioblastoma, pancreas, and colon) failed to show significant advantages for the use of NSAIDs or Coxibs in disease treatment.^50^^,^^51^^,^^52^^,^^53^^,^^54^^,^^55^ Without contradicting the proven role of the PG-mediated role of COX-2 in proliferative diseases, we propose that the AA-induced COX-2 cleavage products present an additional non-catalytic aspect of COX-2 signaling that can modulate (exacerbate, or mitigate) the initiation and/or course of proliferation and should be explored. The conditions used in our study were such that COX inhibitors were applied before AA stimulation, which resulted in a significant reduction of cleavage. This result may explain why the effect of Coxibs in cancer prevention is much more established. In contrast, the use of COX inhibitors after inflammation has already begun may result in the presence of a significant amount of COX-2 fragments, possibly enough to modify the course of the disease. These fragments are no longer sensitive to Coxibs, which may explain the mixed results of the clinical trials that examined treatment at later disease stages. Finally, we also suggest that the presence of COX-2 fragments in tumors is not detected because the primary method of detection in the clinical setting is immunohistochemistry, which is excellent for determining the amount of expression and tissue localization but does not indicate the size of the protein. The use of advanced methods of LC-MS/MS is necessary to identify the COX-2 fragments that are present within tumors to characterize their possible roles in disease etiology.

A non-catalytic role has been ascribed to COX-2 in a series of studies showing that it inhibits the function of the tumor suppressor p53 through a direct interaction between the two proteins.^56^^,^^57^^,^^58^ In contrast to our findings, the effect of COX-2 on p53 was found to occur without stimulation. Furthermore, p53 was shown to interact with the non-catalytic regions of COX-2, while we identify effects that are mediated by regions of the catalytic domain of the protein. Nonetheless, both studies complement each other by supporting additional cellular functions for COX-2, beyond those obtained by the generation of PGs.

In summary, we propose that COX-2 fulfills two different cellular functions that occur in two waves. The first wave is through its enzymatic action and the generation of PGs that activates G protein-coupled receptors and relay their signals through soluble second messengers. The second wave arises from the secession of catalytic activity, which leads to the secretion of part of the inactive F.L. protein by exocytosis^14^ as well as to its controlled proteolysis. The products of proteolysis can then migrate to different cellular compartments where they affect cellular function in mechanisms that are PG-independent. Characterization of these pathways and discovery of additional functions of COX-2 fragments will further our understanding of the complex role of COX-2 in pathologies, such as proliferative diseases and chronic inflammation and may help identify novel targets for intervention against its components.

### Limitations of the study

The main limitation of the study is its reliance on an antibody-based method to detect COX-2 fragments. Most commercially available antibodies are directed against the N- or C-termini epitopes of COX-2, which prevents the identification of fragments that do not contain these regions. This limitation is also relevant to the identification of possible proteolysis of COX-1, which bears a great similarity to COX-2 and catalyzes AA by the same mechanism. However, using commercially available antibodies, we are unable at this time to demonstrate such cleavage. Therefore, to study the additional cleavage points identified by LC-MS/MS, there may be a need to generate custom-made antibodies by immunizing animals with the appropriate immunogenic peptide antibodies against them.

Another limitation is the development of blots using an automated Imager. The automatic exposure is designed so that bands are not overexposed when saturated. In several instances, the formation of the 40 kD COX-2 fragment produces a relatively weak signal that is difficult to visualize in the exposure of the whole blot. To overcome this limitation, we always take two images of the same blot: one of the whole gel, and one that covers the strong signal and captures the differences in the levels of the COX-2 fragment. The places where this was done are stated in the figure legend (Figures 1E and 1G).

Finally, the sample size of the patient cohort (*n* = 8) limits the generalization of the findings to *in vivo* models and they only support the rationale for studying the unknown function of COX-2 fragments.

## Resource availability

### Lead contact

Further information and any requests should be directed to and will be fulfilled by the lead contact, Liza Barki-Harrington (Lbarki@psy.haifa.ac.il).

### Materials availability

All unique/stable reagents generated in this study are available from the lead contact with a completed materials transfer agreement.

### Data and code availability

• The mass spectrometry proteomics data have been deposited to the ProteomeXchange Consortium via the PRIDE partner repository^59^ with the dataset identifier PXD057448. The RNA-seq data have been deposited to the NCBI SRA database with the dataset identifier PRJNA1178884. Both are publicly available as of the date of publication.
• This study does not report an original code.
• Any additional information required to reanalyze the data reported in this paper is available from the lead contact upon request.

## Acknowledgments

The authors wish to thank Dr. Sagie Schif-Zuck from the Flow Cytometry Service Unit, University of Haifa for assistance with the flow cytometry analyses, and Dr. Maya Lalzar from the Bioinformatics Services Unit, University of Haifa, for assistance with transcriptome data. We also thank the Technion Smoler Proteomics Center for their help in running the MS analysis. This work was funded by the 10.13039/501100003977Israel Science Foundation (1445/14 and 2240/19 to L. B.-H. and 1623/17 to O.K.) and by the 10.13039/501100003975Israel Cancer Association (#20210063 to L. B.-H.).

## Author contributions

Conceptualization: S.T., O.K., and L.B.-H.; investigation: L.H. H.-B.., S.T., A.A.E., O.E., R.S.-E., and T.L.; methodology: S.T. and L.B.-H.; formal analysis: L.H. H.-B., S.T., A.A.E., O.E., R.S.-E., O.K., M.M., and L.B.-H.; resources: O.K., M.M., and L.B.-H.; writing—original draft: L.B.-H.; writing—review and editing: L.H. H.-B, S.T., A.A.E., O.K., M.M., and L.B.-H.; funding acquisition: O.K. and L.B.-H.; supervision: L.B.-H.

## Declaration of interests

The authors declare no competing interests.

## STAR★Methods

### Key resources table

**Table:** 

REAGENT or RESOURCE	SOURCE	IDENTIFIER
**Antibodies**		

Rabbit monoclonal anti-COX-2	Cell Signaling Technology	12282 (H5H5) XP, RRID: AB_2571729
Goat polyclonal anti-COX-2 (C-20)	Santa Cruz Biotechnology Inc.	SC-1745, RRID: AB_631309
Rabbit polyclonal anti-Beta Actin	Abcam	ab115777, RRID: AB_10899528
Mouse monoclonal anti GAPDH	Cell Signaling Technology	97166, RRID: AB_2756824
Rabbit monoclonal Lamin B (D9V6H)	Cell Signaling Technology	13435, RRID: AB_2737428
Mouse monoclonal alpha tubulin	Santa Cruz Biotechnology Inc.	SC-398103, RRID: AB_2832217
HRP-conjugated goat anti-rabbit IgG	Jackson ImmunoResearch Laboratories	111-035-144, RRID: AB_2307391
Horseradish peroxidase-conjugated goat anti-mouse IgG	Jackson ImmunoResearch Laboratories	115-035-003, RRID: AB_10015289
Peroxidase AffiniPure™ Bovine Anti-Goat IgG (H + L)	Jackson ImmunoResearch Laboratories	805-035-180, RRID: AB_2340874

**Biological samples**		

Patient-derived colorectal samples	HaEmek Medical Center	Ha’Emek Medical Center- 0049–19

**Chemicals, peptides, and recombinant proteins**		

Acetonitrile HPLC Grade (CAS-75-05-08)	J.T. Baker	9012
Arachidonic acid	Cayman Chemical	90010
Aspirin	Cayman Chemical	70260
Alpha linolenic acid	Cayman Chemical	90210
Albumin, bovine fraction V	MP Biomedicals	160069
Beta-Nicotinamide adenine dinucleotide hydrate	Sigma-Aldrich	N7004
Protein Assay Dye Reagent Concentrate	BioRad	5000006
BLUEYE prestained protein ladder	BIO-HELIX	IMPM007
Calpain inhibitor I (ALLN)	Sigma-Aldrich	11086090001
Calpain inhibitor III -zvf	Sigma-Aldrich	208722
Cholera toxin	Sigma-Aldrich	C8052
DMEM medium	Sartorius	A01-052-1
DMEM/F12 medium	Sartorius	1-170-01A
Docosahexaenoic Acid (DHA)	Cayman Chemical	90310
Complete Protease Inhibitor Cocktail	Merck Millipore	11697498001
DAPT (GSI-IX)	Cayman Chemical	13197
Deoxycholic acid	Sigma	D2510
E64	Sigma Aldrich	E3132
EDTA sodium salt	J.T. Baker	8993–01
Epidermal growth factor	Thermo Fischer Scientific	AF115
Eicosapentaenoic Acid (sodium salt)	Cayman Chemical	21908–5
F12 Nutrient Mixture (Ham’s)	Sartorius	01-095-1A
Formic acid 99% ULC/MS	Bio-Lab ltd	691414400
Fetal bovine serum	Rhenium	10270106
Horse serum	Sigma	H1138
HY-MYCOPLASMA DETEC 24T PCR KIT+INT.CON	HY labs	KI5034I
Hydrocortisone	Sigma	H0888
Ibuprofen	Cayman Chemical	16793
Insulin	Sigma	I9278
Imperial™ Protein Stain Coomassie dye	Rhenium	24615
Iodonitrotetrazolium chloride	Sigma-Aldrich	I8377
L-lactate dehydrogenase (LDHA)	Sigma-Aldrich	10127230001
L-lactate (sodium)	Sigma-Aldrich	PHR1113
Lipopolysaccharide (LPS)	Sigma Aldrich	L6529
Linoleic acid	Sigma-Aldrich	L1012
Methoxy-PMS	BioTag	HY-D0937
MitoTracker™ Dyes for Mitochondria Labeling	Thermo Fischer	M7512
NAD-Mitochondrial Membrane Potential	Abcam	ab113852
Nitrocellulose blotting membrane- 0.45 μm	Tamar	980045
Nonidet P-40 substitute	Thermo Scientific	J19628.500
Palmitic Acid	Cayman Chemical	10006627
PBS	Biological Industries	02-023-5A
PD 15606	Sigma Aldrich	D5946
Phenylmethanesulfonyl fluoride	Sigma	066K0720
PolyJet *In Vitro* DNA Transfection Reagent	SingaGen	SL100688
Prostaglandin E2	Cayman Chemical	14010
Prostaglandin G2	Cayman Chemical	17010
Prostaglandin H2	Cayman Chemical	17020
Protein Assay Dye Reagent Concentrate	Bio-Rad	5000006
Protein A/G PLUS-Agarose	SantaCruz Biotechnology	SC-2003
RNase-ExitusPlus	Sartorius	01-897-1B
RNA save	Sartorius	01-891-1B
Sodium fluoride	Sigma Aldrich	201154
Tris base, DNase RNase protease free, electrophoresis tested	Fisher Bioreagents	BP152
Trypsin EDTA Solution A	IMBH	L0932-100
Trypsin, sequencing grade modified	Promega	V5111

**Critical commercial assays**		

NEBNext Ultra RNA library Prep kit for Illumina	Thermo Fischer Scientific	E75030L
Prostaglandin E2 Parameter Assay Kit	R&D Systems, Inc.	KGE004B
Prostaglandin E2 ELISA kit-Monoclonal	Cayman Chemical	514010
QuikChange Lightning Site-Directed Mutagenesis Kit	Agilent	210519
PureYield™ Plasmid Midiprep System	Promega	A2492
Quick-RNA™ Miniprep Plus Kit	Zymo	ZR-R1058
NEBNext Ultra RNA library Prep kit for Illumina	Thermo Fischer Scientific	E75030L

**Deposited data**		

Raw RNA-seq transcriptome data	This paper	NCBI SRA database Bioproject PRJNA1178884
Raw proteomics data	This paper	PRIDE partner repository^59^ Accession PXD057448

**Experimental models: Cell lines**		

Human A549	ATCC	CCL-185
Human Caco-2	ATCC	HTB-37
Human HCT-116	ATCC	CCL-247
Human HEK 293 passage 13-40	ATCC	CRL-1573
Human HeLa	ATCC	CRM-CCL2
Human MCF10A	ATCC	CRL-10317
Murine RAW 264.7	ATCC	TIB-71
Human U87	ATCC	HTB-14

**Recombinant DNA**		

Plasmid: G533A COX-2	Rowlinson et al.^4^	N/A
Plasmid:	This paper	N/A
Plasmid	This paper	N/A

**Software and algorithms**		

*Adobe Illustrator CS5.1*	*Adobe Community*	www.adobe.com, RRID: SCR_010279
Adobe Photoshop CS5.1	Adobe Community	www.adobe.com, RRID: SCR_014199
EndNote X7	Endnote	www.endnote.com, RRID: SCR_014001
GraphPad Prism 10	GraphPad software	www.graphpad.com, RRID: SCR_002798
ImageQant TL 1D v8.1	Cyntiva	www.cytivalifesciences.com, RRID: SCR_014246
MSFragger version 4.0	Kong et al.^60^	FragPipe version 21.1 (https://fragpipe.nesvilab.org/)

**Other**		

Empore C18 for StageTip	CDS Analytical	2215
Fused-Silica capillaries, tube with emitter tip without frit, 360umOD x 75umID x 8um Tip.	MSWIL	ICT36007508-50-5

### Experimental Model and study participant details

#### Cell cultures

A549 lung cancer cells were grown in F12 media. RAW 264.7, U87, HeLa, HEK 293, HCT-116 and Caco-2 cells were all grown in DMEM. All media was supplemented with 10% heat-inactivated fetal bovine serum, and 100 U/ml penicillin. MCF-10A cells were grown in DMEM/F12 supplemented with 5% donor horse serum, 20 ng/mL epidermal growth factor (EGF), 10 μg/mL insulin, 0.5 μg/mL hydrocortisone, 100 ng/mL cholera toxin, 100 μg/mL streptomycin, and 100 U/ml penicillin.^60^ All cells were grown at 37°C in a humidified atmosphere of 95% air, and 5% CO_2_. All lines were tested for mycoplasma contamination and were found clean. None of the lines were authenticated.

#### Patient samples

Eight Human colon biopsies were obtained from individuals diagnosed and operated on at Ha’Emek Medical Center, Afula Israel. Patients were 18–80 years old both male and female that were diagnosed with adenocarcinoma of the colon or rectum, with tumors that were graded at least B2 or higher, as determined by pathology reports. Biopsies were obtained during surgery upon written consent from patients according to the Helsinki regulations (Ha’Emek Medical Center- 0049–19). They were placed in microfuge tubes containing 1 mL RNA save (Biological Industries) and flash-frozen in liquid N_2_ pending analysis. Two samples were obtained from each patient: one from the tumor and one from what was considered a clean margin, and the data regarding COX-2 expression and intensity were compared between the normal and tumor samples of each patient. The association of sex or gender to the results cannot be determined due to the small sample size.

### Method details

#### cDNA constructs

All constructs were generated in the laboratory using the primers listed in Table S7, using the QuikChange Lightning Site-Directed Mutagenesis (Agilent Technologies, Santa Clara, CA, USA), according to the Manufacturer’s instructions. Cloning was verified at the core sequencing facilities of Hylabs (Rehovoth, Israel).

#### Transfection with plasmid cDNA

HEK 293 cells were seeded a day before transfection Transient transfections were carried out in sub-confluent monolayers (70–80%) using PolyJet (SignaGen Laboratories) at a ratio of 1:3 cDNA: PolyJet, according to the manufacturer’s instructions.

#### Cell stimulation, processing, and immunoblotting

Cells were stimulated with different concentrations of AA for 30 min. All other fatty acids used in the study were applied at 20 μM for 30 min before AA stimulation, as were the inhibitors ibuprofen (200 μM) and aspirin (100 μM). Treatments with the different protease inhibitors were applied for 18–24 h at the following concentrations: PD15606 (50 μM), ALLN (10 μM), E64 (10 μM), zVF (50 or 100 μM).

Following stimulation, monolayers were washed twice with ice-cold PBS and lysed in RIPA/SDS buffer (50 mM Tris pH- 8, 150 mM NaCl, 5 mM EDTA, 1% v/v NP-40, 0.5%, 0.5% w/v deoxycholic acid, 0.1% w/v SDS, 10 mM NaF, 10 mM sodium-pyro-phosphate and Complete Protease Inhibitor cocktail tablets with 0.1 mM PMSF). Lysates were spun at 17,000 x g for 10 min at 4^o^C, the pellets were removed and protein concentrations were determined (BioRad Protein Assay).^30^ Samples were prepared at concentrations of 1 mg/mL in 5X Laemmeli buffer and sonicated on ice for 10–15 s at an amplitude of 15 of the Fisherbrand Model 50 Sonic Dismembrator. 30–50 μg of total protein lysates were used in all blots. Nitrocellulose membranes containing the immuno-complexes or total cell lysate proteins were incubated with primary antibodies at a dilution of 1:500–1000. Proteins were visualized by a WesternBright ECL (Advansta) and quantified using the Amersham Imager 600 (GE) and quantified using ImageQant TL 1D v8.1 software.

Subcellular fractionation was done using the Rapid, Efficient And Practical (REAP) method.^61^ HEK293 cells grown as monolayers in 10 cm diameter dishes were washed in ice-cold PBS, scraped, collected in 1.5 mL micro-centrifuge tubes in 1 mL of ice-cold PBS, and centrifuged for 10 s using a tabletop centrifuge. Supernatants were discarded cell pellets were resuspended in 900 μL of ice-cold PBS supplemented with 0.1% NP-40, 0.1 mM PMSF, and Complete Protease Inhibitor cocktail, and triturated 5 times. 300 μL of the lysate was removed and labeled as “total lysates". 100 μL of 4 × Laemmli sample buffer were added and the sample was kept on ice until the sonication step. The remaining sample was centrifuged at 8000 × g at 4^O^C for 2 min and the supernatant was collected and labeled as "Non-nuclear (Non-nuc)". The remaining pellet was suspended in ice-cold PBS with 0.1% NP40 and centrifuged twice at 8000 × g at 4°C for 2 min. The supernatant was discarded and the pellet was resuspended in ice-cold 0.1% NP-40, 0.1 mM PMSF, and Complete Protease Inhibitor cocktail tablets and labeled as the "Nuclear fraction (Nuc)". All fractions except for the Non-Nuc were sonicated twice for 5 s each using Fisherbrand Model 50 Sonic Dismembrator**.**

#### Immunoprecipitation and mass spectrometry

7 x 10^6^ RAW 264.7 cells were grown in 15 cm dishes and stimulated overnight with 100 ng/mL LPS, followed by a 30-min stimulation with 50 μM AA. The reaction was terminated by two subsequent washes with ice-cold PBS and lysis in 0.5 mL RIPA/SDS buffer. Samples were centrifuged at 17,000 × g at 4°C for 10 min, the supernatants were collected, and protein levels were determined. 100 μg of the lysates were used for total protein assessment. 3–3.5 mg protein were used for IP experiments. Samples were pre-cleared with 20 μL of protein A/G beads for 60 min, centrifuged again at 17,000 × g at 4°C for 2 min and the remaining supernatant was used for overnight immunoprecipitation with 2 mg goat polyclonal anti-COX-2 (Santa Cruz Biotechnology, SC-1745) and 30 μL protein A/G beads with continuous rotation at 4°C. After three subsequent washes with 0.75 mL RIPA/SDS, lysates were separated by a 10% sodium dodecyl sulfate (SDS) polyacrylamide gel. The entire protein lane of each sample was cut into three horizontal gel pieces and processed by in-gel trypsin digestion procedure,^62^ followed by peptides desalting using C18 StageTip.^63^ Whole proteome and activity-guided proteasome profiling samples were analyzed using a Q-Exactive Plus mass spectrometer (Thermo Fisher) coupled to Easy nLC 1000 or Dionex (Thermo Fisher). The peptides were resolved by reverse-phase chromatography on 0.075 × 180 mm fused silica capillaries (J&W) packed with Reprosil reversed-phase material (Dr. Maisch; GmbH, Germany). Peptides were eluted with a linear gradient of 6–28% acetonitrile 0.1% formic acid for 60 min followed by a 15 min gradient of 28–95% acetonitrile 0.1% formic acid and a 15 min wash at 95% acetonitrile with 0.1% formic acid in water (at flow rates of 0.15–0.2 μL/min).

The MS analysis was performed in positive mode using a range of m/z 300–1800, resolution of 70,000 for MS1 and 17,500 for MS2, using, repetitively, full MS scan followed by HCD of the 10 most dominant ions selected from the first MS scan with an isolation window of 1.8 m/z. Other settings used were as follows: a dynamic exclusion of 20 s, NCE = 27, minimum AGC target = 8x10^3^, and intensity threshold = 1.3x10^5^. Data analysis was done using MSFragger version 4.0^64^ via FragPipe version 21.1 (https://fragpipe.nesvilab.org/). In all searches, the raw files were searched against the Uniprot Mouse/Human proteome (Downloaded on Feb 2024 and includes 54940/20413 protein sequences, respectively). MSFargger searches were performed by using semi-tryptic digestion. Search criteria included precursor and fragments tolerance of 20 ppm with oxidation of methionine, and protein N-terminal acetylation set as variable modifications.

#### COX-2 activity measurements

The media of 3 × 10^5^ transfected HEK 293 in 6-well dishes was siphoned, and the cells were washed twice with warm PBS and incubated in 1 mL of serum-free DMEM with AA for 30min. In the fatty acid experiments, cells were treated with the different compounds or with the appropriate vehicle for 30 min, followed by an additional 30 min with 50 μM AA. At the end of the experiment, the supernatant was collected and the levels of PGE_2_ were analyzed using Prostaglandin E_2_ ELISA kits, according to the manufacturer’s instructions.

#### Mitochondrial activity measurements

0.05 x 10^6^ HEK 293 cells were seeded in 12-well dishes and transfected with 0.75 μg F.L. COX-2 or fragment cDNA. 72 h post-transfection the ΔΨm was measured.^24^ The cells were dissociated with trypsin and washed twice with ice-cold 0.2% BSA in PBS. After centrifugation for 5 min at 1500 rpm, the cells were incubated in the dark at 37°C, for 20 min with MitoTracker for Mitochondria Labeling (Ex/Em 490/516) for general mitochondria staining, and TMRE for mitochondrial membrane potential (Ex/Em 549/575 nm). After an additional wash with 1 mL of 0.2% BSA, the cells were resuspended in 300 μL 0.2% BSA and immediately analyzed by the BD FACSCanto II flow cytometer with DACSDiva software (BD Biosciences). Gates were set to exclude necrotic cells and cellular debris and the fluorescence intensity of events within the gated regions was quantified. Data were collected from 10000 events for each sample. The relative percentage of cells with high Δψm was calculated as % high Δψm of total (high Δψm + low Δψm).

#### L-lactate level measurements

L-lactate levels were measured by a colorimetric assay of lactate dehydrogenase (LDH) activity 0.3 x 10^6^ HEK 293 cells were seeded in 6-well dishes and transfected with 1 μg cDNA of F.L. COX-2 or fragments in 1 mL complete DMEM media. 72 h post-transfection the external media of the cells was collected and probed for the presence of lactate in triplicates.^65^ 50 μL of sample supernatants were placed in a flat-bottom 96-well dish followed by the addition of 50 μL Assay buffer (200 mM Tris-Base, pH = 8.2, 2.2 mg/mL β-NAD, 0.5 mg/mL INT, 1 μg/mL L-LDH, 6 μg/mL 1-Methoxy-PMS) and incubated for 1 h at room temperature in the dark. The reaction was terminated by the addition of 50 μL Acetic Acid (1 M) and the absorbance was measured at 490 nm-ref. 650 nm with a microplate absorbance reader. If lactate concentration was above that of the standard curve, samples were diluted in DMEM.

#### Proliferation assays

For live cell tracking experiments, 3 × 10^4^ HEK 293 cells were seeded into 24-well dishes and transfected the next day as described above. Plates were placed in the IncuCyte 5XS live-cell analysis system (Essen Bioscience, Ann Arbor, MI, USA) for 48 h and snapshots were taken every 30 min. Percent confluence was analyzed over time using the IncuCyte SX5 G/O/NIR Optical module software at the Bioimaging Unit, University of Haifa. Each experimental condition contained four biological repeats. Data of each condition was normalized to that value at time zero (first reading) to control for technical variability of the replicates.

### Quantification and data analysis

#### Transcriptome analyses

Total RNA was prepared from four biological replicates of transiently transfected HEK 293 cells using the Quick-RNA MiniPrep kit (Cat. # ZR-R10554, Zymo Research). Library preparation was performed using the NEBNext Ultra RNA library Prep kit for Illumina (Cat. #E7530L, Thermo Fischer Scientific, Waltham, MA USA), according to the manufacturer’s protocol. Sequencing (single-read, 50bp) was carried out using the Illumina HiSeq 2500 at the TGC-Technion Genome Center (Technion, Haifa, Israel). Trimming of poly-A and poly-G leftovers was done using the PRINSEQ tool (https://sourceforge.net/projects/prinseq/files/) removing reads with more than 58% of A or G in twain with a 3′ tail filter (poly-A) of more than 8 bases. Qualified reads were later trimmed for adaptors and low-quality reads using Trimmomatic (v0.39; http://www.usadellab.org/cms/index.php?page=trimmomatic). In addition, reads were clipped to a size between 40 and 60 bases for optimal mapping. Gene expression levels were quantified using Htseq-count (0.6.1-py2.7) and filtered for genes covered by at least 10 reads in all samples. Differential expression was analyzed using EdgeR (3.2.4). *p*-values were corrected for multiple testing using the Benjamini-Hochberg method, and differential expression was considered significant for adjusted *p*-value <0.05. The differentially expressed (DE) genes were subjected to gene set enrichment analysis using Metascape (accessed March 2024^22^). Cutoff for significant enrichment was adjusted for multiple testing (Benjamini Hochberg) and the resulting q-values are reported (Tables S5 and S6).

#### Statistical analysis

Statistical analyses were done using the GraphPad Prism Software 10.3.1. Normality tests (D’Agostino & Pearson and Shapiro-Wilk) confirmed that all data is normally distributed. Statistical significance was determined by t-test or one-way ANOVA according to group numbers. Post-hoc analysis was performed with Dunnett’s multiple comparisons post-test when appropriate. *p* values <0.05 were considered significant. ^∗^*p* < 0.05, ^∗∗^*p* < 0.01, ^∗∗∗^*p* < 0.001, and ^∗∗∗∗^*p* < 0.0001.

## Supplemental information

Document S1. Figures S1–S3 and Table S7

Table S1. Genes with a significant increase in expression in CT1-compared to CT2-expressing cells, related to Figure 5

Table S2. Genes with a significant decrease in expression in CT1-compared to CT2-expressing cells, related to Figure 5

Table S3. Genes with a significant increase in expression in CT1-compared to CT2-expressing cells and no change between CT2-expressing cells and untransfected cells, related to Figure 5

Table S4. Genes with a significant decrease in expression in CT1-compared to CT2-expressing cells and no change between CT2-expressing cells and untransfected cells, related to Figure 5

Table S5. Gene sets showing significant enrichment among genes upregulated in CT1-compared to CT2-expressing cells and not changing between CT2-expressing cells and untransfected cells, related to Figure 5

Table S6. Gene sets showing significant enrichment among genes downregulated in CT1-compared to CT2-expressing cells and not changing between CT2-expressing cells and untransfected cells, related to Figure 5
