# Supplementary material for: Activity-dependent COX-2 proteolysis modulates aerobic respiration and proliferation in a prostaglandin-independent manner
Source: iScience. 2024 Nov 17;27(12):111403. doi: 10.1016/j.isci.2024.111403 (PMC11647142; doi:10.1016/j.isci.2024.111403)
Supplement: Document S1. Figures S1–S3 and Table S7 [file mmc1.pdf]

iScience, Volume *27*

Supplemental information

Activity-dependent COX-2 proteolysis modulates aerobic respiration and proliferation in a prostaglandin-independent manner

Liat Hagit Hartal-Benishay, Sharon Tal, Amal Abd Elkader, Omar Ehsainieh, Ranin Srouji-Eid, Tali Lavy, Oded Kleifeld, Martin Mikl, and Liza Barki-Harrington

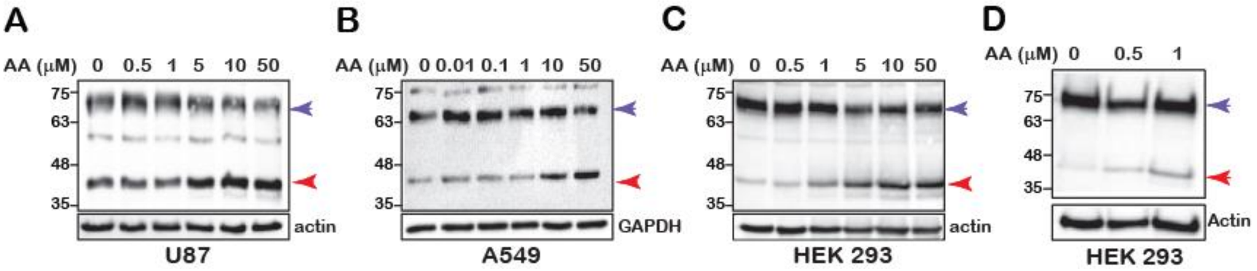

Figure S1: The appearance of the COX-2 fragment is dose-dependent, related to Figure 1. Representative immunoblots of (A) U87 (human brain glioma, endogenous COX-2 expression) (B) A549 (human lung adenocarcinoma, endogenous COX-2 expression) (C-D) HEK 293 (human embryonic kidney, transfected) were treated with the indicated concentrations of AA for 30 min.

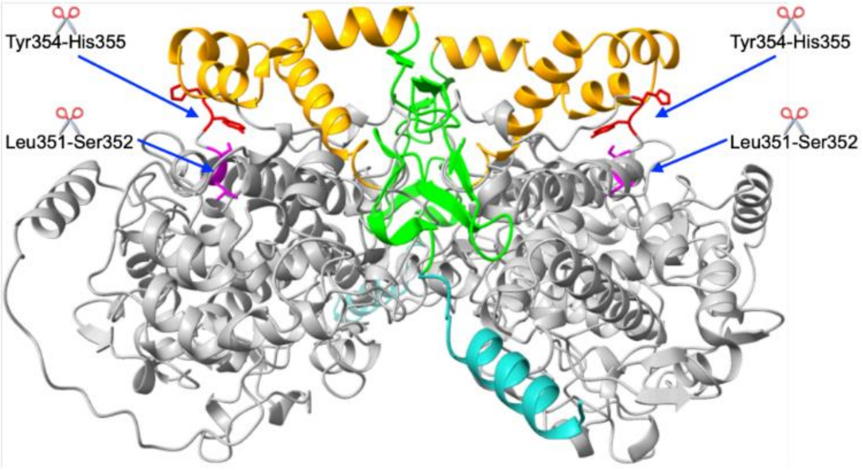

Figure S2: A 3-D structure of COX-2 depicting the two cleavage sites identified in the study, related to Figure 4.
The signal sequence is colored in light blue, the EGF domain in green, the MDB domain in orange, and the catalytic domain is depicted in grey. The first cleavage site is shown in pink and the second cleavage is in red. The numbering on the cleaved residues is based on the COX-1 sequence.

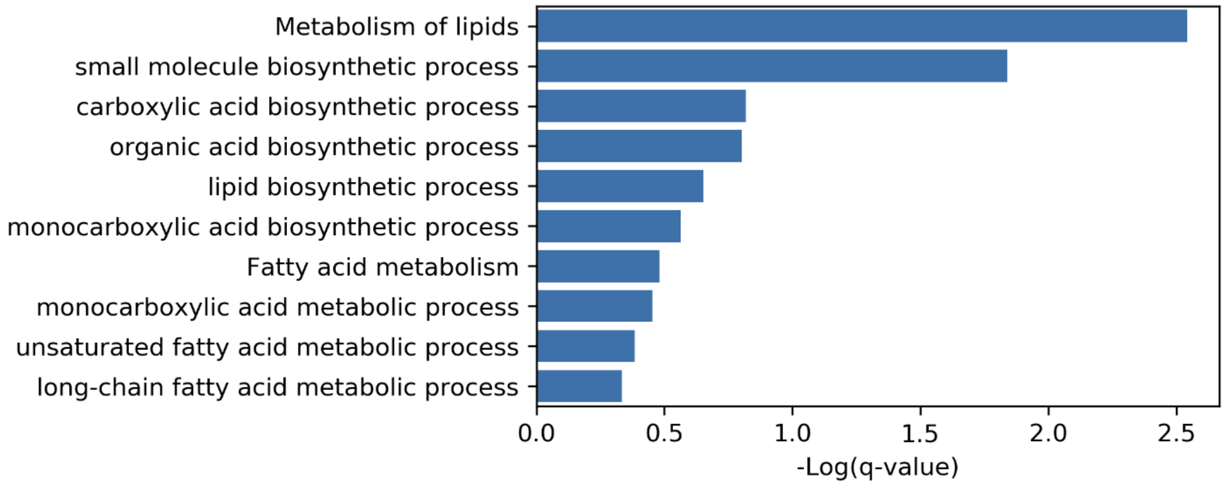

Figure S3: Gene set enrichment analysis for genes with reduced expression in CT1-expressing cells, related to Figure 5.

Top 10 most significantly enriched functional groups (as determined using Metascape) among the downregulated genes in CT1- compared to CT2-expressing cells, which showed no significant change between CT2-expressing and control cells.

Name	Clone	Primers	Template
Mut 1	L352A S353A Y355A H356A F	gtgattgaagattatgtgcaacacgcggctggctatgccgccaaactgaaatt	F.L. COX-2
Mut 1	L352A S353A Y355A H356A R	aatttcagtttggcggcatagccagccgcgtgttgcacataatcttcaatcac	F.L. COX-2
Mut 2	G354A on Mut 1 F	cagtttgaaggcagcggcactcaagtgttgcac	Mut 1
Mut 2	G354A on Mut 1 R	gtgcaacacttgagtgccgctgccttcaaactg	Mut 1

Table S7: List of primers used for cloning, related to STAR methods
